# OP17 Updated Cost-Effectiveness Threshold Estimates For 174 Countries Based On Projected Growth In Life Expectancy, Health Expenditure, And Gross Domestic Product

**DOI:** 10.1017/S0266462325100755

**Published:** 2025-12-29

**Authors:** Andres Pichon-Riviere, Michael Drummond, Sebastian Garcia Marti, Federico Augustovski

## Abstract

**Introduction:**

In 2023, we published widely cited cost-effectiveness thresholds (CETs) for 174 countries. This update refined our approach by incorporating gross domestic product (GDP) growth projections to adjust target increases in life expectancy and health expenditure. This methodology provides more precise estimates tailored to the economic and health contexts of individual countries, enabling better informed resource allocation decisions.

**Methods:**

We revised our 2023 methodology by integrating International Monetary Fund projections of GDP growth for the next five years to establish customized targets for life expectancy (LE) and health expenditure (HE) growth. This update accommodates countries where the prior assumption of median growth within income groups was less applicable. The CETs were derived so that the effect of new interventions on the evolution of LE and HE is set within predefined goals. To provide guidance on CETs, we projected country-level HE and LE increases by income level based on World Bank data to ensure the estimates align with individual country realities.

**Results:**

The updated thresholds yielded values consistent with our 2023 estimates, yet notable differences emerged in countries with distinct GDP growth trajectories or unique health spending patterns. For example, thresholds for India and similar countries adjusted upward to reflect ambitious yet realistic targets for HE and LE growth. Across 174 countries, most thresholds remained below one GDP per capita, confirming their relevance for guiding economic evaluations. These nuanced updates demonstrate the flexibility and applicability of our approach in diverse contexts, particularly for low- and middle-income countries.

**Conclusions:**

By incorporating GDP growth projections, this study enhanced the precision of cost-effectiveness thresholds, ensuring their applicability across varying country contexts. These updates provide critical guidance for health systems aiming to balance efficiency, equity, and sustainability, particularly in resource constrained settings.

